# Mitomycin-C for HPV-Positive and HPV-Negative Platinum-Refractory, Recurrent or Metastatic Head and Neck Squamous Cell Carcinoma: A Phase 2 Trial

**DOI:** 10.3390/cancers17213568

**Published:** 2025-11-04

**Authors:** Peter Oppelt, Jessica Ley, Christine Auberle, Brendan Knapp, Jesse Zaretsky, Fei Wan, Douglas Adkins

**Affiliations:** 1 Alvin J. Siteman Cancer Center, St. Louis, MO 63110, USA; poppelt@wustl.edu (P.O.); auberlec@wustl.edu (C.A.); bjknapp@wustl.edu (B.K.); jzaretsky@wustl.edu (J.Z.); dadkins@wustl.edu (D.A.); 2 Division of Medical Oncology, Washington University School of Medicine, 660 South Euclid, Campus Box 8056, St. Louis, MO 63110, USA; 3 Division of Public Health Sciences, Biostatistics Shared Resource, Alvin J. Siteman Cancer Center, Washington University School of Medicine, St. Louis, MO 63110, USA; wan.fei@wustl.edu

**Keywords:** mitomycin-C, p53, head and neck squamous cell carcinoma, recurrent disease, metastatic disease, HPV-positive

## Abstract

**Simple Summary:**

The primary aim of this phase 2 trial was to determine the objective response rate (ORR) with mitomycin-C among patients with human papillomavirus (HPV)-positive (cohort A) and HPV-negative (cohort B) platinum-refractory, recurrent or metastatic head and neck squamous cell carcinoma (RM-HNSCC). The ORR was 9.1% in cohort A and 0% in cohort B. We concluded that mitomycin-C had limited activity in HPV-positive, and no activity in HPV-negative, platinum-refractory RM-HNSCC.

**Abstract:**

Background/Objectives: Functional p53 is critical for anti-tumor activity of mitomycin-C. In wild-type *TP53* human papillomavirus (HPV)-positive squamous cell carcinoma (SCC) cell lines, mitomycin-C repressed E6 oncoprotein expression and induced p53, p21, and Bax, resulting in apoptosis. In mutant *TP53* HPV-negative SCC cell lines, mitomycin-C was inactive. The primary aim of this trial was to determine the objective response rate (ORR) with mitomycin-C among patients with HPV-positive (cohort A) and HPV-negative (cohort B) platinum-refractory, recurrent or metastatic head and neck SCC (RM-HNSCC). Methods: Patients with platinum-refractory RM-HNSCC received mitomycin-C (10 mg/m^2^ on day one every five weeks) until discontinuation criteria were met. Tumor response was assessed by RECIST1.1. We hypothesized an ORR of ≥30% (H_1_) with mitomycin-C (vs. H_0_ ORR of ≤10%). Using a two-stage Simon phase 2 design for each cohort, 2 or more responses among 12 evaluable patients were required to enroll 23 additional patients. H_1_ was accepted if 6 or more responses occurred among 35 evaluable patients (power 0.90; one-sided α = 0.10). Results: Forty-seven patients were treated with mitomycin-C: 34 in cohort A and 13 in cohort B. Tumor response occurred in 3 of 33 evaluable patients in cohort A (ORR 9.1%, 95%CI: 0–19.4) and in 0 of 12 evaluable patients in cohort B. The duration of tumor responses in cohort A was 2.3, 2.5, and 4.5 months. The most common treatment-related AEs of any grade were anemia (96%), fatigue (62%), and thrombocytopenia (40%). No treatment-related deaths occurred. Conclusions: Mitomycin-C had limited activity in HPV-positive, and no activity in HPV-negative, platinum-refractory RM-HNSCC.

## 1. Introduction

Over 800,000 new cases of head and neck squamous cell carcinoma (HNSCC) occur each year [1]. After curative-intent therapy, half of these patients will develop recurrent or metastatic (RM) disease. When our trial began in 2015, the standard first-line therapy for RM-HNSCC included a platinum agent, 5-fluorouracil, and cetuximab (the EXTREME regimen) [2]. A taxane agent was often given as second-line therapy. In platinum-refractory disease, docetaxel resulted in an objective response rate (ORR) of 12% [3,4]. Subsequent studies showed that the PD-1 inhibitors pembrolizumab or nivolumab resulted in ORRs of 13–16% in platinum-refractory disease, and first-line therapy with pembrolizumab given with or without platinum-based chemotherapy improved overall survival (OS) compared to the EXTREME regimen [3,4,5,6]. However, disease progression eventually occurs with each of these treatment options. Additional strategies are needed to treat RM-HNSCC.

Mitomycin-C is an alkylating agent that causes DNA adduct formation. Functional p53 protein is critical for anti-tumor activity of mitomycin-C. In cancer cells with wild-type *TP53*, mitomycin-C increases p53 expression resulting in p53-mediated apoptotic cell death [7,8]. However, cancer cells with loss-of-function mutations in *TP53* are resistant to mitomycin-C [9]. Inactivation of p53 is a frequent somatic event in HNSCC and occurs by two key mechanisms. In human papillomavirus (HPV)-positive oropharynx (OP) SCC, the E6 oncoprotein results in proteasome degradation of p53 [10]. In contrast, mutations in the *TP53* gene occur in most cases of HPV-negative HNSCC [11]. In HPV-positive SCC cell lines, mitomycin-C repressed E6 expression and induced p53, p21, and Bax, resulting in apoptosis; however, in HPV-negative SCC cell lines with mutant *TP53*, mitomycin-C was inactive [12,13].

Mitomycin-C was first used to treat patients with cancer in 1958 [14,15]. Clinical trials conducted before the discovery of the etiologic link between HPV and cancer showed that mitomycin-C resulted in tumor response rates of 18–21% in patients with RM-HNSCC [14,15,16,17], and mitomycin-C, a potent radiosensitizer, improved outcomes when combined with radiation therapy to treat locally advanced HNSCC [15,18,19]. After the emergence of cisplatin, use of mitomycin-C in patients with HNSCC stopped. Since then, evidence showed that mitomycin-C was an active agent in several HPV-induced SCCs, including those of the anus, cervix, penis, and vulva [15,20,21]. In anal cancer treated with chemoradiation, local–regional control and OS were better with mitomycin-C compared to cisplatin [20]. In metastatic cervical cancer, mitomycin-C resulted in tumor response rates of 12–27% [15].

In the modern era, there are no studies that report the efficacy of mitomycin-C in patients with RM-HNSCC, specifically among cohorts of patients with HPV-positive or HPV-negative disease. In this trial, we investigated the efficacy of mitomycin-C in patients with HPV-positive (cohort A) and HPV-negative (cohort B), platinum-refractory RM-HNSCC. Because p53 function can be induced by genotoxic stress in HPV-positive cancers, we hypothesized that mitomycin-C would be an active agent in HPV-positive RM-HNSCC. Because somatic mutant *TP53* occurs in 84% of cases of HPV-negative HNSCC [11], we hypothesized that mitomycin-C would have limited activity in HPV-negative disease. Herein, we report the results of this trial.

## 2. Materials and Methods

### 2.1. Patient Population

Eligible patients were 18 or older with incurable RM-HNSCC of the oropharynx, larynx, hypopharynx, oral cavity, or upper (level 2–3) neck nodes without a known primary tumor site. Incurable disease was defined as local or regional recurrence or distant metastases, not amenable to complete resection or definitive radiation therapy. Measurable disease, defined by Response Evaluation Criteria in Solid Tumors (RECIST) v1.1, was required [22]. Initially, the protocol required disease progression on a platinum agent, 5-fluorouracil, cetuximab, and a taxane (given as monotherapy or in combination) used to treat RM-HNSCC. After pembrolizumab and nivolumab were approved to treat RM-HNSCC, the eligibility criteria were amended in October 2020 to require prior disease progression on a platinum agent and a PD-1 inhibitor given to treat RM-HNSCC. Other inclusion criteria included Eastern Cooperative Oncology Group (ECOG) performance status 0–3 and adequate marrow and organ function (absolute neutrophil count [ANC] ≥ 1000/mcl; platelets ≥ 75,000/mcl; total bilirubin ≤ 1.5 mg/dL; AST, ALT, and alkaline phosphatase ≤ 2.5× institutional upper limit of normal [IULN]; creatinine ≤ IULN or creatinine clearance ≥ 60 mL/min/1.73 m^2^). Key exclusion criteria included active brain metastases or other serious active malignancy.

Tests required to determine eligibility included are as follows: physical examination, complete blood count, metabolic panel, and computerized tomography (CT) scans. In SCC of the oropharynx or level 2–3 neck nodes without a known primary tumor site, immunohistochemistry was performed to assess expression of the p16 protein, a surrogate biomarker of HPV [23]. HPV-positive HNSCC was defined as SCC of the oropharynx or level 2–3 neck nodes without a known primary tumor site that had nuclear and cytoplasmic staining for p16 present in ≥70% of tumor cells. HPV-negative HNSCC was defined as SCC of the oral cavity, larynx, or hypopharynx, and SCC of the oropharynx or upper neck nodes without a known primary tumor site that had staining for p16 present in <70% of tumor cells. The Washington University institutional review board approved the protocol (201503060) on 9 April 2015, and all study participants provided written informed consent to participate. The Washington University quality assurance and scientific monitoring committee performed independent data monitoring. This trial is registered with ClinicalTrials.gov, NCT02369458, on 23 February 2015.

### 2.2. Procedures

Mitomycin-C 10 mg/m^2^ was administered intravenously on day one of each five-week cycle. On day two of each cycle, pegfilgrastim 6 mg was administered subcutaneously. Mitomycin-C continued until disease progression, intolerable adverse event (AE), patient/physician decision to discontinue study drug, or withdrawal from the trial. Physical examinations, blood counts, and a metabolic panel were performed on days 1 and 15 of each cycle. AEs were monitored on days 1 and 15 of each cycle and graded using the National Cancer Institute Common Toxicity Criteria for Adverse Events (NCI-CTCAE) v3.0. Adequate marrow and organ function, as previously defined, were required to administer mitomycin-C. If the laboratory requirements were inadequate on day one, mitomycin-C was held and the patient was seen weekly with repeat blood work. The dose of mitomycin-C was reduced by 25% if the nadir after the previous cycle was ANC 500–1000/mcL or platelet count 20,000–50,000/mcL, and by 50% if the nadir after the previous cycle was ANC < 500/mcL or platelet count < 20,000/mcL. Dose re-escalations were not permitted. Mitomycin-C was discontinued if hemolytic uremic syndrome (HUS) occurred.

Tumor response assessments were performed after every two cycles by contrast-enhanced neck and chest CT scan (as well as abdominal/pelvis CT scans, if applicable to sites of disease). Tumor response was assessed by the investigator using RECIST1.1 (i.e., complete response [CR], partial response [PR], stable disease [SD], and progressive disease [PD]) [22].

### 2.3. Outcomes

The study included cohort A (patients with HPV-positive disease) and cohort B (patients with HPV-negative disease). The primary endpoint for each cohort was ORR, defined as the proportion of patients that achieved an objective tumor response (CR or PR) with mitomycin-C. Secondary endpoints for each cohort included progression-free survival (PFS: time from cycle one day one to progression or last follow-up), OS (time from cycle one day one to death or last follow-up), and duration of response (DoR: time from documentation of CR or PR until disease progression or last follow-up). The proportion of patients with AEs was summed across both cohorts. Follow-up was defined as the time from cycle one day one to last follow-up.

Testing for *TP53* was not widely clinically available when the study began in 2015 but became available in subsequent years. Thus, in some patients, tailored genome sequencing of tumor tissue was performed to assess for mutations in *TP53.* Methods for sequencing included in-house testing [24], FoundationOne^®^CDx (Cambridge, MA, USA), Caris MI^®^ Profile (Irving, TX, USA), OncoDNA^®^ (Gosselies, Charleroi, Belgium), and Tempus xT^®^ (Chicago, IL, USA). An exploratory endpoint was the ORR with mitomycin-C in patients with wild-type *TP53* cancer or mutant *TP53* cancer.

### 2.4. Statistical Analyses

The primary hypothesis was that mitomycin-C would result in tumor responses in a clinically relevant proportion of patients with HPV-positive (cohort A) and HPV-negative (cohort B) RM-HNSCC. An ORR of 30% (H_1_) in each cohort warranted further study, and an ORR of ≤10% (H_0_) did not. Using a two-stage Simon design for each cohort, two or more responses among 12 evaluable patients in the first stage were required to expand to the second stage and accrue an additional 23 evaluable patients. Six or more tumor responses among 35 evaluable patients would conclude efficacy in that cohort. Up to 35 evaluable patients were to be accrued to each cohort, for a total sample size of up to 70 patients. The probability of termination after the first stage was 0.66 if the true ORR was 10% (power 0.90; one-sided α = 0.10).

Patients who had undergone at least one tumor response assessment were evaluable for the primary objective of ORR. Patients that had not undergone a tumor response assessment were non-evaluable for the primary objective of ORR, and an equivalent number of patients were added to the sample size to meet the evaluability target. All patients who received at least one dose of study drug were evaluable for PFS, OS, and assessment of AEs. We calculated the proportion of patients with objective tumor response (CR and PR). Median PFS and OS with 95%CIs were estimated by the Kaplan–Meier (KM) method [25]. Chemotherapy delivery (number of planned doses administered, total dose/m^2^) was summarized using proportions with medians and ranges. AEs were summarized by type and grade using proportions and 95%CIs.

Investigators conducted the collection, analysis, and interpretation of the data, and preparation of the manuscript. All authors had access to all data in the study. The corresponding author had final responsibility for the decision to submit for publication.

## 3. Results

### 3.1. Trial Profile

**Figure 1:**
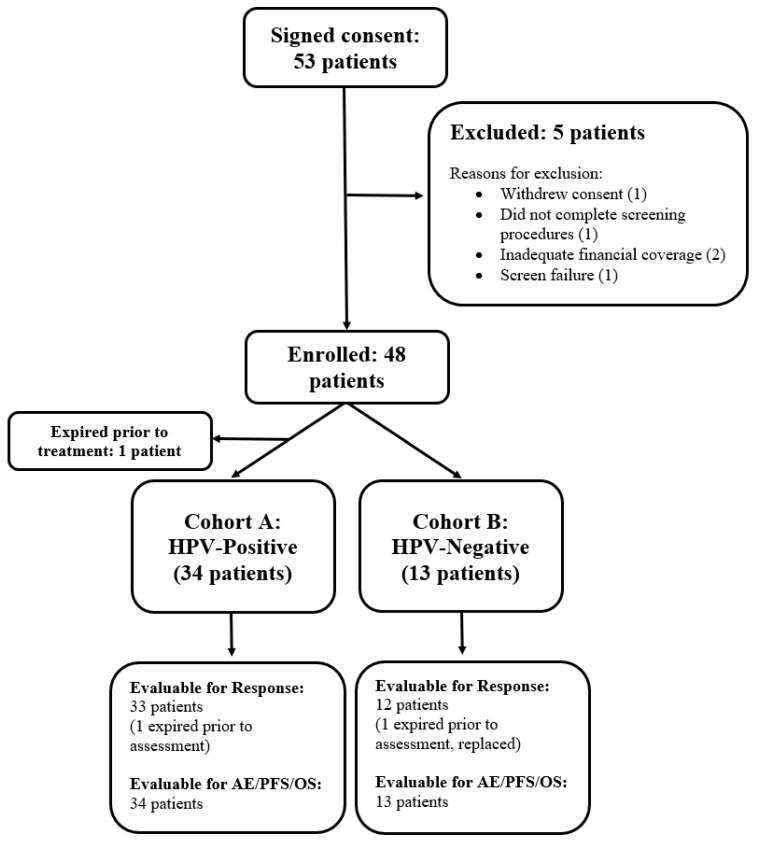
Trial profile. Of the 53 patients who signed consent, 47 patients enrolled in the trial and received the study drug (34 in cohort A and 13 in cohort B).

Between 19 May 2015 and 14 March 2022, 48 patients were enrolled in the trial: 35 in cohort A (HPV-positive RM-HNSCC) and 13 in cohort B (HPV-negative RM-HNSCC) (Figure 1). One patient in cohort A withdrew from the trial before administration of study drug and was not evaluable for any endpoint. Thirty-three patients in cohort A and 12 patients in cohort B were evaluable for the primary endpoint of tumor response. Thirty-four patients in cohort A and 13 patients in cohort B were evaluable for the secondary endpoints of AEs, PFS, and OS.

The cut-off date for data analysis presented here was 2 November 2024. Median follow-up of the patients in cohort A was 6.6 months (IQR: 2.7–12.0) and in cohort B was 3.2 months (IQR: 1.5–9.4).

### 3.2. Patient and Tumor Characteristics

Baseline characteristics are shown in Table 1. Most patients were older males, had a history of tobacco, and an ECOG performance status of 0 or 1. Thirty-six patients (76.6%) were enrolled based on the original eligibility criteria of disease progression on a platinum agent, 5-FU, cetuximab, and a taxane agent, and 11 patients (23.4%) were enrolled based on the amended eligibility criteria of disease progression on a platinum agent and a PD-1 inhibitor. In the latter group, three of the patients also met the original eligibility criteria.

### 3.3. Objective Response Rate

Criteria to proceed from Simon’s first stage to the second stage of the trial were met in cohort A, but not in cohort B. Accrual to cohort A was terminated early (after 33 evaluable patients) due to futility, based on the limited number of tumor responses observed in those patients.

**Figure 2:**
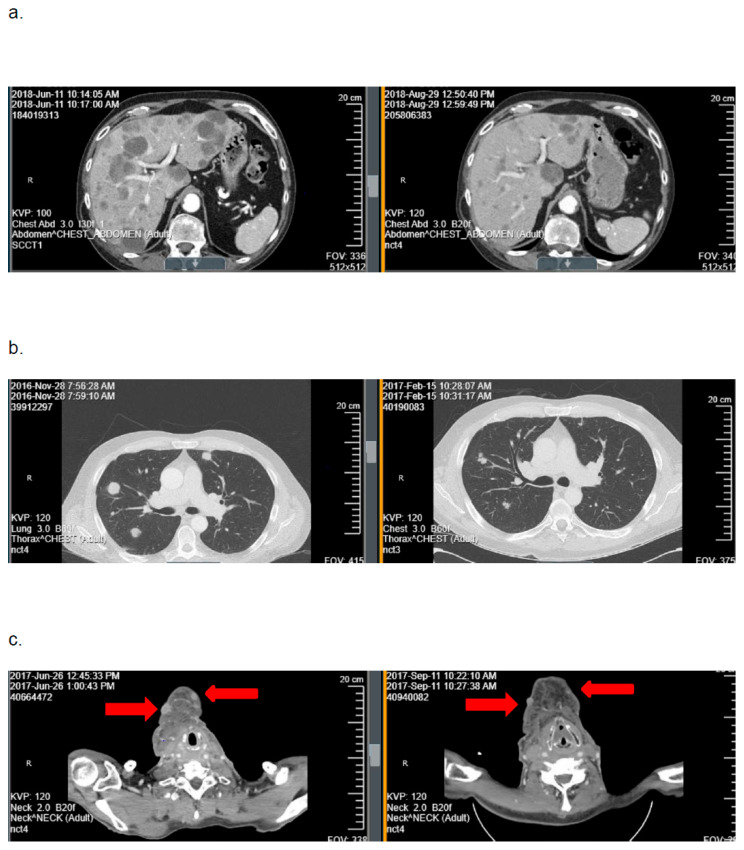
Representative examples of computed axial tomography imaging scans obtained at baseline (**left**) and after two cycles of mitomycin-C (**right**) from the three patients on cohort A with a tumor response with mitomycin-C: (**a**) patient 21: hepatic metastases, (**b**) patient 10: pulmonary metastases, and (**c**) patient 14: subcutaneous metastases.

**Figure 3:**
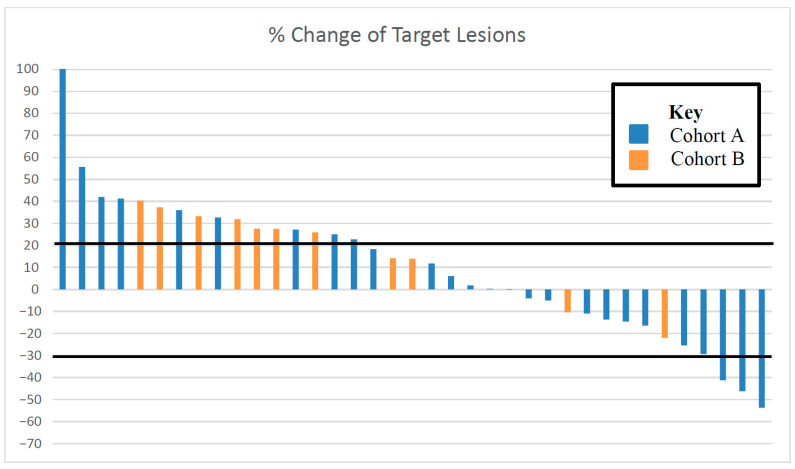
Best percent change in target lesions among 26 evaluable patients in cohort A (blue) and the 12 evaluable patients in cohort B (orange). Bold black lines delineate −30% to +20%.

In cohort A, the ORR was 9.1% (95%CI: 0–19.4). Best tumor response included PR in 3 patients (9.1%), SD in 10 (30.3%), and PD in 20 (60.6%). One response was confirmed and two responses were unconfirmed (Figure 2). As seen in Figure 3, the depths of these partial responses were significant. The DoR’s were 2.3, 2.5, and 4.5 months. In cohort B, the ORR was 0%. The best tumor response included SD in three patients (25%) and PD in nine (75%).

### 3.4. TP53 Mutational Status and Tumor Response

Mutant *TP53* occurred in the cancers of 2 of 27 evaluable patients (7.4%) in cohort A and 9 of 10 evaluable patients (90%) in cohort B. The remaining patients did not have genomic sequencing performed.

Across both cohorts, tumor response occurred in 2 of 26 evaluable patients with wild-type *TP53* cancers and 0 of 10 evaluable patients with mutant *TP53* cancers. The third patient who experienced a tumor response did not have genome sequencing performed.

### 3.5. Progression-Free Survival and Overall Survival

**Figure 4:**
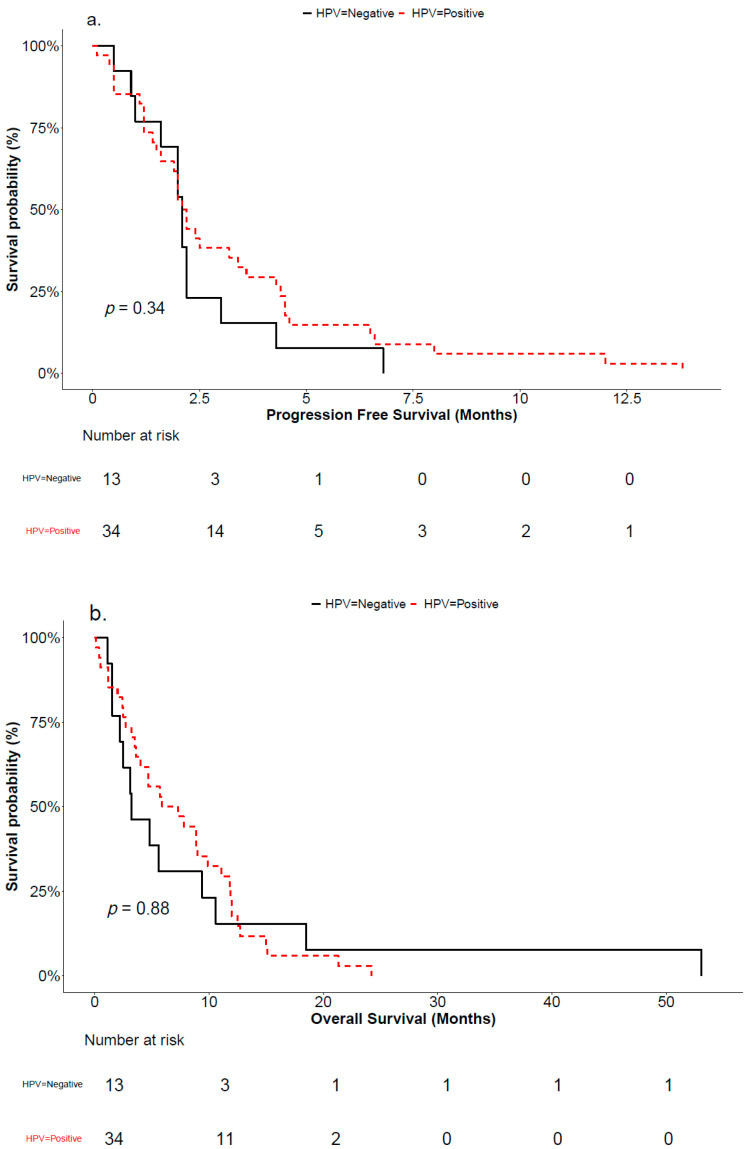
(**a**) Progression-free survival (PFS) and (**b**) overall survival (OS) for patients in cohort A (red curve) and cohort B (black curve).

PFS and OS are summarized in Figure 4. In cohort A, the median PFS was 2.2 months (95%CI: 1.5–3.4) and the median OS was 6.6 months (95%CI: 3.5–9.9). At the last follow-up, all 34 patients had expired. Causes of death included disease progression (22 patients), co-morbidity (2), complication of disease (9), or unknown (1).

In cohort B, the median PFS was 2.1 months (95%CI: 1.0–2.2) and the median OS was 3.2 months (95%CI: 1.5–9.4). At the last follow-up, all 13 patients had expired. Causes of death included disease progression (10 patients), co-morbidity (2), or complication of disease (1).

Seven patients in Cohort A were not included due to malignant pleural effusion obstructing lung lesions in two, expired on study before scans in three, and no CT scans at discontinuation in two. One patient in Cohort B was not included, as progression was unmeasurable due to the sheer disease burden. Three patients with −30% to +20% change in target lesions had progression: two had increase in non-target lesions and one had clinical progression.

### 3.6. Treatment Delivery and Adverse Events

The median number of mitomycin-C cycles administered per patient in cohort A was two (IQR: 1–4) and in cohort B was two (IQR: 1–2). Dose modifications did not occur in either cohort. Dose holds occurred in two patients, both in cohort A, each due to mild (grade 2) transient thrombocytopenia. The most common treatment-related AEs of any grade were anemia (96%), fatigue (62%), and thrombocytopenia (40%) [Table 2].

## 4. Discussion

In this trial, the primary hypothesis of achieving an ORR of ≥30% with mitomycin-C was not met in either cohort. The ORR was 9.1% among patients with HPV-positive, platinum-refractory RM-HNSCC (cohort A) and 0% among patients with HPV-negative, platinum-refractory disease (cohort B). Although three patients in cohort A experienced deep tumor responses with mitomycin-C (Figure 2 and Figure 3), the DoRs were brief (≤4.5 months). Based on these data, further investigation of mitomycin-C as monotherapy in patients with biomarker-unselected, platinum-refractory RM-HNSCC is not warranted.

A key finding of this trial was that the ORR with mitomycin-C in patients with platinum-refractory RM-HNSCC was surprisingly low, particularly in HPV-positive disease. The pre-clinical science and the efficacy of mitomycin-C across a broad array of HPV-induced SCCs led us to hypothesize a higher level of activity of mitomycin-C among patients with HPV-positive RM-HNSCC [10,15,20,21]. It is not clear why the ORR in cohort A was lower than expected. It is possible that mechanisms of resistance to prior therapies (especially platinum agents) may be cross-mechanisms of resistance to mitomycin-C, that pre-clinical models do not accurately reflect the more complex biology and environment of a clinical tumor that has been molded by several prior therapies, and that the clinical activity of mitomycin-C in HPV-positive cancers from other sites may not be generalizable to HNSCC. Cohort B (patients with HPV-negative, platinum-refractory disease) was included in this trial for the following three reasons: (1) to validate the pre-clinical science which suggested that mitomycin-C would be inactive in mutant *TP53* disease, (2) patients with HPV-negative, wild-type *TP53* disease may have benefited from mitomycin-C, and (3) clinical trials conducted before the discovery of the etiologic link between HPV and cancer showed that mitomycin-C resulted in tumor response rates of 18–21% in patients with RM-HNSCC [14,15,16,17]. As expected, no responses were observed in cohort B, although the sample size was small.

We set a high-bar (ORR ≥ 30%) for the primary hypothesis, which, in retrospect, may have been overly optimistic since 39 of the 45 patients (87%) evaluable for response had previously experienced disease progression on a platinum agent, 5-FU, cetuximab, and a taxane agent, and all patients had platinum-refractory disease. In such heavily pretreated patients, a lower bar for success may have been more appropriate.

We divided patients in our trial into two cohorts based on HPV status, with the expectation that nearly all patients in cohort A would have wild-type *TP53* disease and 84% of patients in cohort B would have mutant *TP53* disease [11]. The results of the trial are in line with those expectations. Among the evaluable patients, wild-type *TP53* occurred in the cancers of 92.6% of patients in cohort A and mutant *TP53* occurred in the cancers of 90% of patients in cohort B. Patient selection to include only wild-type *TP53* disease may have yielded different results in cohort B, but likely not in cohort A. Studies to evaluate the activity of mitomycin-C in wild-type *TP53,* HPV-negative disease may be warranted.

Several strategies may improve the tumor response rate with mitomycin-C in patients with RM-HNSCC. Proteasome inhibition sensitized cervical cancer cells to mitomycin-C by preventing degradation of mitomycin-C-induced FasL [26]. These data point to combinations of proteasome inhibitors and mitomycin-C to treat HPV-positive RM-HNSCC. Also, tumor-suppressor miRNAs such as miR-34a, mir-449a, and miR-16 in the setting of DNA damage can regulate apoptosis, senescence, and autophagy in HPV-positive cell lines independent of p53 [27]. In cells lacking functional p53 protein, mitomycin-C adducts induced caspase-independent (non-apoptotic) cell death by depletion of Chk1 and activation of PARP [9]. Mitomycin-C in combination with drugs that target programmed necrosis and autophagy may be useful in HPV-positive RM-HNSCC and HPV-negative RM-HNSCC with mutant *TP53* [28]. Different schedules of mitomycin-C may also improve the efficacy of this drug. We administered mitomycin-C on day one of each five-week cycle. The long cycle interval may permit disease progression to occur between treatment cycles. Mitomycin-C is toxic to marrow stem and early progenitor cells, resulting in delayed blood count recovery. The five-week schedule was utilized to permit recovery of blood counts between cycles. However, schedules whereby the drug is administered at shorter cycle intervals are feasible and may improve the ORR and DoR in patients with RM-HNSCC [15,16,17].

Overall, mitomycin-C was safe to administer to patients with platinum-refractory RM-HNSCC, although we recognize the adverse impact of the high burden of manageable toxicities on patient’s quality of life. Mild (grades 1–2) AEs of any cause occurred in all patients. However, severe (grades 4–5) treatment-related AEs did not occur. Dose reductions due to AEs did not occur, treatment delays were infrequent, and no patients discontinued therapy due to AEs. Hemolytic uremic syndrome, a rare serious AE related to mitomycin-C, did not occur. Allergic reactions to mitomycin-C were not observed in this trial, although they have been reported to occur [29,30].

Several limitations of this trial exist. The study was a phase 2 trial without a direct, parallel comparison group. We enrolled 13 patients into cohort B, and only one of those evaluable patients had wild-type *TP53* cancer. It is possible that a study with a larger sample of patients with wild-type *TP53* HPV-negative disease would reveal that this subset may respond to mitomycin-C [11]. An independent radiologist did not assess tumor response. We did not perform tumor biopsies at enrollment to confirm the status of the *TP53* gene immediately before beginning the study drug. Strengths of the study include the strong pre-clinical and clinical rationale for the potential efficacy of mitomycin-C in subsets of patients with this disease and the prospective trial design with a specific primary hypothesis.

## 5. Conclusions

This trial showed that mitomycin-C had limited activity among patients with HPV-positive, platinum-refractory RM-HNSCC, and was inactive in HPV-negative disease. These results do not warrant further investigation of mitomycin-C as monotherapy in patients with biomarker-unselected, platinum-refractory RM-HNSCC.

## Figures and Tables

**Table 1:** Baseline patient and tumor characteristics *.

Characteristic	Cohort A: HPV-Positive (*n* = 34)	Cohort B: HPV-Negative (*n* = 13)
Age (years) Median	61	61
Range	45–81	54–68
Sex		
Male	33	8
Female	1	5
ECOG Performance Status		
0	20	5
1	11	7
2–3	3	1
Tobacco History		
Yes	25	12
No	9	1
Primary Site		
Oropharynx	32 ^	0
Larynx	0	5
Oral Cavity	0	5
Hypopharynx	0	2
Occult Primary/Upper Neck	2	1
Node		
Site(s) of RM Disease		
Local/Regional	0	2
Distant	22	6
Both	12	5
Prior Therapy for RM Disease		
Platinum/5-FU/Cetuximab/Taxane **	23	13
Platinum/PD-1 Inhibitor ***	11	0

* for the 47 evaluable patients (those treated with the study drug). ** original eligibility criteria used through September 2020. ECOG= Eastern Cooperative Oncology Group. RM = recurrent or metastatic. 5-FU = 5-fluorouracil. *** amended eligibility criteria used after October 2020. Note that some patients in this group may have also been treated with 5-FU, cetuximab, and/or a taxane agent. ^ one patient in cohort A was initially coded as oropharynx SCC; however, upon careful review during the analysis, this patient was determined to have p16 (HPV)-positive larynx SCC. Using the intent-to-treat principle, this patient was included in cohort A.

**Table 2:** Adverse events *.

	Grade 1	Grade 2	Grade 3	Grade 4	Grade 5
Alkaline Phosphatase	14	2	1		
Anorexia	6	6			
Back Pain	3	3			
Bilirubin	3	2	2		
Cardiovascular: Carotid Artery					1 ^a^
Chest Wall Pain			1		
Constipation	5	3	1		
Cough	9				
Creatinine	6	2			
Death NOS					1 ^a^
Dehydration		1	2		
Diarrhea	7				
Dysphagia	10	5	3		
Dyspnea	7	9	4		
Edema: Head and Neck	1	1	1		
Edema: Limb	5	1			
Fatigue	10	17	2		
Headache		1	1		
Hemoglobin	24	17	4		
Hepatitis			1		
Hypercalcemia	5	1	1		
Hyperglycemia		4	1		
Hyperkalemia	3		1		
Hypertension	4	4	1		
Hypoalbuminemia	15	11	1		
Hypocalcemia	7	6			
Hypokalemia	5		3		
Hyponatremia	16	1	4		
Hypophosphatemia		3	1		
Infection with Normal ANC		6	4	2 ^a^	
Leukopenia	5	2			
Lymphopenia	1	17	22	2 ^a^	
Muscle Weakness	1	1	2		
Nausea	8	3			
Neuropathy: Sensory	4	4			
Neutropenia	1	1	1		
Obstruction of Airway					1 ^a^
Pericardial Effusion				1 ^a^	
Platelets	13	6			
Pleural Effusion (Non-malignant)			2		
Pneumothorax			1		
Respiratory Failure				1 ^a^	1 ^a^
Restrictive Cardiomyopathy			1		
AST	9	2		1 ^a^	
ALT	4	1	1	1 ^a^	
Thrombosis/Thrombus/Embolism			2		
Tumor Pain	1	11	2		
Weight Loss	4	1	2		
Xerostomia	7	1	1		

* grade 1–2 adverse events (AEs) with an incidence of ≥10% are shown. All grade 3–5 events are shown. Attribution of AE to mitomycin-C: ^a^ unrelated

## Data Availability

Individual participant data that underlie the results reported in this article, after de-identification (text, tables, figures, and appendices), and the study protocol will be shared beginning 9 months and ending 24 months following article publication with investigators whose proposed use of the data has been approved by an independent review committee (“learned intermediary”) identified for this purpose. Types of acceptable analyses include approved proposal or individual participant data for meta-analyses. Proposals may be submitted up to 24 months following article publication. Information regarding submitting proposals and accessing data may be submitted to jcley@wustl.edu.

## Abbreviations

5-FU	5-Flurouracil
AE	adverse event
ALT	alanine aminotransferase
ANC	absolute neutrophil count
AST	aspartate aminotransferase
CI	confidence interval
CR	complete response
CT	computerized tomography
dL	deciliter
DNA	deoxyribonucleic acid
DoR	duration of response
ECOG	Eastern Cooperative Oncology Group
HNSCC	head and neck squamous cell carcinoma
HPV	human papillomavirus
HUS	hemolytic uremic syndrome
IQR	interquartile range
IULN	institutional upper limit of normal
KM	Kaplan–Meier
m2	meter squared
mcL	microliter
mg	milligram
min	minute
miRNA	micro ribonucleic acid
mL	milliliter
NCI-CTCAE	National Cancer Institute Common Toxicity Criteria for Adverse Events
NOS	not otherwise specified
OP	oropharynx
ORR	objective response rate
OS	overall survival
PARP	poly(ADP-ribose) polymerase
PD	progressive disease
PFS	progression-free survival
PR	partial response
RECIST	response evaluation criteria in solid tumors
RM	recurrent or metastatic
RNA	ribonucleic acid
SCC	squamous cell carcinoma
SD	stable disease
TP53	tumor protein 53
